# Determination of selected cetyltrimethylammonium halide parameters by molecular modeling. Study of their adsorption on montmorillonite

**DOI:** 10.1186/1758-2946-6-S1-P1

**Published:** 2014-03-11

**Authors:** Bekhelifa Leila, Zizi Zahia, Hallouch Mustapha, BenAhmed Abdellatif

**Affiliations:** 1 Laboratory of Materials and Catalysis, Chemistry Department, Faculty of Sciences, Hai larbi Ben M'hidi BP 89 University of Sidi- Bel – Abbes; SIDI BEL ABBES, 22000, Algeria

## 

There are new chemical substances that increase the strength of washing products such as surfactants. However, these products are not biodegradable and can all be found in surface water, groundwater. Cationic surfactants are one of the pollutants of greatest danger to man is his environment. Therefore, we have determined some parameters of three cationic surfactants by molecular modeling, using the CHEM 3D bio. We, therefore, optimized geometries and minimized the energy of cetyltrimethylammonium chloride (CTAC), the cetyltrimethylammonium bromide (CTAB) and cetyltrimethylammonium iodide (CTAI) by molecular mechanics. we calculated the size of these molecules and their molecular partition coefficients and their ovalities using molecular dynamics. The results showed that the three pollutants are adsorbed on Mt (natural), Mt (Mg) and Mt (Ca) and are not adsorbed on Mt (Na). Mt (K) can adsorb only CTAC. The values of the partition coefficient of the three surfactants are equal to unity. The cationic surfactants studied are highly hydrophilic and spend very little through a membrane. Al so, these values showed that these surfactants are much more soluble in water than in octanol. Both surfactants, CTAB and CTAI have the highest ovality values. They can therefore more easily approach a spheric or cylindric form. CTAC has the lowest ovality, it presents more difficulty to approach a spherical or cylindrical shape.

